# A New Aortic Arch Dissection Classification: The Fuwai Classification

**DOI:** 10.3389/fcvm.2021.710281

**Published:** 2021-09-14

**Authors:** Juntao Qiu, Xinjin Luo, Jinlin Wu, Wei Pan, Qian Chang, Xiangyang Qian, Xiaogang Sun, Bo Wei, Liang Zhang, Shen Liu, Wenxiang Jiang, Cuntao Yu

**Affiliations:** ^1^Department of Cardiovascular Surgery, National Center for Cardiovascular Diseases, Fuwai Hospital, Chinese Academy of Medical Sciences, Peking Union Medical College, Beijing, China; ^2^Department of Anesthesia, Baylor College of Medicine, Texas Heart Institute, Houston, TX, United States; ^3^Department of Cardiovascular Surgery, Institute of Heart, Lung and Blood Vessel Disease, Beijing Anzhen Hospital, Capital Medical University, Beijing, China; ^4^Department of Cardiovascular Surgery, Peking University International Hospital, Beijing, China

**Keywords:** aortic arch dissection, classification system, clinical characteristic, mortality, surgery

## Abstract

**Aims:** We describe a new aortic arch dissection (AcD) classification, which we have called the Fuwai classification. We then compare the clinical characteristics and long-term prognoses of different classifications.

**Methods:** All AcD patients who underwent surgical procedures at Fuwai Hospital from 2010 to 2015 were included in the study. AcD procedures are divided into three types: Fuwai type Cp, Ct, and Cd. Type Cp is defined as the innominate artery or combined with the left carotid artery involved. Type Cd is defined as the left subclavian artery or combined with the left carotid artery involved. All other AcD surgeries are defined as type Ct. The Chi-square test was adopted for the pairwise comparison among the three types. Kaplan-Meier was used for the analysis of long-term survival and survival free of reoperation.

**Results:** In total, 1,063 AcD patients were enrolled from 2010 to 2015: 54 patients were type Cp, 832 were type Ct, and 177 were type Cd. The highest operation proportion of Cp, Ct and Cd were partial arch replacement, total arch replacement, and TEVAR. The surgical mortality in type Ct was higher compared to type Cd (Ct vs. Cd = 9.38 vs. 1.69%, *p* < 0.01) and type Cp (Ct vs. Cp = 9.38 vs. 1.85%, *p* = 0.06). There was no difference in surgical mortality of type Cp and Cd (*p* = 0.93). There were no significant differences in the long-term survival rates (*p* = 0.38) and free of aorta-related re-operations (*p* = 0.19).

**Conclusion:** The Fuwai classification is used to distinguish different AcDs. Different AcDs have different surgical mortality and use different operation methods, but they have similar long-term results.

## Introduction

Aortic dissection is a life-threatening medical emergency with a 10% to 20% risk of in-hospital mortality (1–3). The DeBakey and Stanford classifications are the most commonly used methods to categorize aortic dissection. However, these two classifications have unclear definitions for aortic arch dissection (AcD), especially when the distal aortic arch is involved. Von Segesser firstly defined the distal aortic arch dissection as non-A-non-B dissection (4). This naming method was not widely accepted, and the therapeutic strategy did not reach a conclusion, partly because there was no unified definition.

Aortic arch surgery is complicated and high risk. Partial arch replacement and total arch replacement techniques have different prognoses (5). However, the traditional classification was not able to respond to operation methods for AcD patients. At present, various new aortic arch operation technologies are emerging, such as hybrid technique, endovascular thoracic branched stent, etc (6). There should be a classification corresponding to operation methods for AcD patients. Therefore, we propose the Fuwai classification for AcD to distinguish differences in aortic arch involvement and guide in the selection of an operation method (7).

This study aimed to compare the clinical characteristics and long-term prognosis in different types of AcD. We also explored whether the new aortic dissection classification could help in the selection of operation methods.

## Methods

### Fuwai Classification of Aortic Arch Dissection

**Figure 1:**
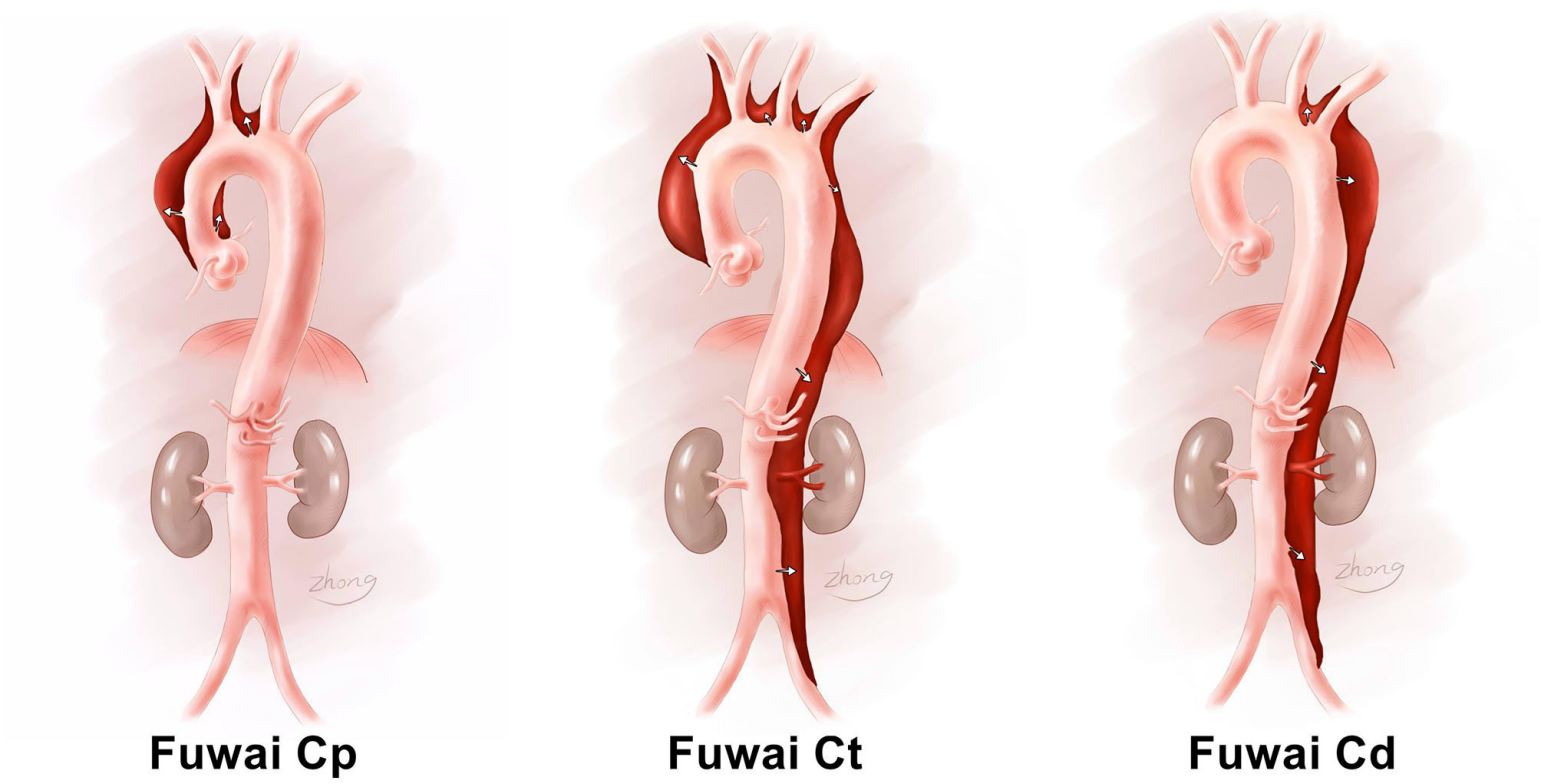
Schematic illustration depicting Fuwai classification.

The Fuwai classification is based on dissection propagation. The Fuwai classification subdivides the aortic arch into the proximal aorta, distal aorta, and total aorta. The proximal aortic arch is the proximal end of the innominate artery and includes the distal end of the left common carotid artery. The distal aortic arch is the proximal end of the left common carotid artery and includes the distal end of the left subclavian artery. If the aortic dissection only involves the proximal arch, it is denoted as Fuwai type Cp (p is short for proximal). If the aortic dissection only involves the distal arch, it is denoted as Fuwai type Cd (d is short for distal). Except for these two classifications, all other AcDs are denoted as Fuwai type Ct (t is short for total or transverse). The Fuwai classification for aortic dissection is illustrated in Figure 1.

### Patients and Collection

The research, as part of our registered project in the Chinese Clinical Trial Registry (ChiCTR1800015338), is being reported in line with Strengthening the Reporting of Observational Studies in Epidemiology (STROBE). The study was approved by the Chinese ethics committee, with informed consent not required due to its observational nature (Reference Number: ChiECRCT-20180041).

The data in this study were obtained from the aortic dissection patients of Fuwai hospital. All clinical patient data with aortic dissection were collected from January 2010 to December 2015. Patients were selected based on the following inclusion and exclusion criteria. Inclusion criteria were as follows: (1) patients with aortic dissection who had accepted and received surgical treatment; (2) preoperative and intraoperative pathologic identification of aortic dissection; (3) patients who underwent aortic dissection between January 2010 and December 2015; (4) the involvement of the aortic arch. Exclusion criteria were as follows: (1) the patient or family members refused surgical treatment; (2) imaging diagnosis of suspicious aortic dissection, but no definite diaphragm and false lumen found during surgery; (3) the aortic arch was not involved. A total of 1,063 patients were enrolled in this study.

The comorbidities and postoperative complications were defined according to the Society of Thoracic Surgeons definitions, which is available online at http://www.sts.org/nationaldatabase (detailed description of the definition in the study can be seen in the Supplementary Material).

### Therapeutic Strategy

**Figure 2:**
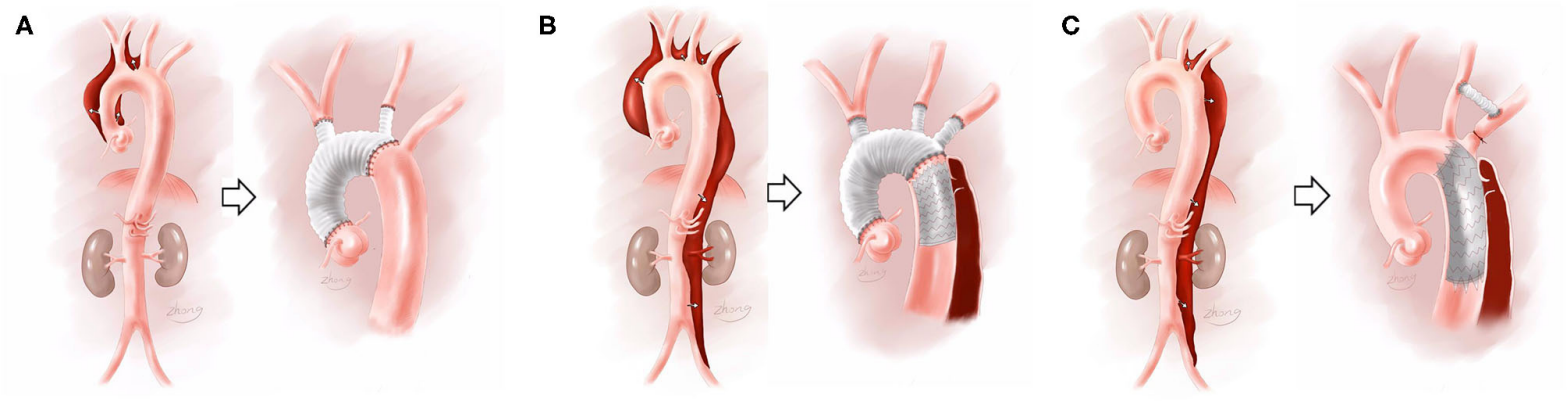
Fuwai classifications and their corresponding surgical treatment. **(A)** Referred to Fuwai Cp and its corresponding partial aortic arch replacement; **(B)** referred to Fuwai Ct and its corresponding total aortic arch replacement + stent trunk surgery; **(C)** referred to Fuwai Cd and its corresponding TEVAR+ debranch surgery.

All patients with aortic dissection who were admitted to our center were prioritized for diagnosis and treatment. Emergency surgery was adopted for Fuwai type Cp or Ct when the ascending aorta was involved. For Fuwai type Cd, we would perform the operation if there were complications in the patient's situation, but we otherwise adopted conservative medical treatments. We performed different operations, according to the Fuwai classification. The main surgical method for the different types is shown in Figure 2. For Fuwai Cp patients, a partial aortic arch replacement was primarily performed. If the patient had an aortic arch dilatation or presented with intramural hematoma, aortic ulcer, or aortic constriction, the lesioned aorta was replaced with an artificial graft, and total aortic arch replacement was performed. If the patient had connective tissue disease, such as Marfan syndrome, the scope of the artificial graft was expanded based on the aortic lesion to prepare for secondary surgery, and total aortic arch replacement plus frozen elephant trunk was performed. When we performed partial arch replacement, femoral artery-right atrium was usually used for cardiopulmonary bypass (CPB) building, and hypothermic circulatory arrest (HCA) was not needed as we were able to cross-clamp the aorta on the arch. When it was necessary to perform traditional total aortic arch replacement, HCA was used, as we could not cross clamp the aorta during distal vascular anastomosis. For Fuwai Ct aortic dissection, a traditional total aortic arch replacement was performed. The axillary artery–right atrium space was usually used for CPB building, and HCA was also applied. If the dissection involved the descending aorta, stents were employed. If the patient was older than 50 and had severe pre-surgical complications, a hybrid total aortic arch replacement was considered. A hybrid total aortic arch replacement was performed under moderate hypothermic CPB. For Fuwai Cd dissection, a debranched approach was employed. For example, the left common carotid and left subclavian artery bypass method was performed, and subsequently thoracic endovascular aortic repair (TEVAR) was performed. For partial chronic Fuwai Cd aortic dissection, where the true lumen was small and the false lumen was large, TEVAR could not repair lesion aorta completely, and total thoracic and abdominal aortic replacement was considered (The detailed description of surgery selection was shown in Supplemental Material-therapy).

### Follow-Up

All surgical survivors were followed up at 3 months, 1, 3, and 5 years after discharge, mainly via telephone questionnaire. Outpatient information was gathered on survival aorta-related re-operation.

### Statistical Analysis

Statistical analysis was performed using SAS 9.3 statistical software (SAS Institute, Cary, NC). For continuous variables, normal distribution analysis was initially performed. If the normal distribution was present, the single-factor variance analysis was adopted. If the data were not normally distributed, the Fisher test and the median and quartiles were used. For classification variables, the chi-square test was adopted. For the three procedure types, pairwise comparison was used. Kaplan-Meier was used to analyze patients' long-term survival and survival free of reoperation related to aortic surgery. The primary endpoints of the study were surgical mortality and major operations. The secondary endpoints were long-term survival and survival free of reoperation.

## Results

### General Characteristics

From 2010 to 2015, there were 1,063 patients enrolled: 54 patients were type Cp, 832 patients were type Ct, and 177 patients were type Cd. The average age of 1,063 patients was 48.62 ± 11.26 years, with 818 male patients (76.95%). Of the total patient population, 46 patients (4.33%) had a partial aortic arch replacement, 865 patients (81.37%) had a total aortic arch replacement, and 125 patients (11.76%) had TEVAR. Among the 1,063 patients, surgical mortality was 7.71%. Post-operative neurological damage occurred in 127 patients, accounting for 11.94% (2.26% had cerebral infarction, 3.76% paraplegia occurred, and the remaining patients suffered from mental disorders, accounting for 5.93%). A total of 981 patients who survived surgery were followed up, with 15 (1.53%) lost during the follow-up period. The median follow-up time was 28 months, the shortest period was 9 months and the longest was 81 months. The overall survival rate was 97.1, 95.4, and 94.7% after 1, 3, and 5 years, respectively. There were 35 patients (3.29%) who required reoperation related to aortic surgery. Of the total patient population, 97.66, 96.34, and 95.04% did not require re-operation at the first, third, and fifth years, respectively.

### Pairwise Comparison of Various Aortic Arch Dissection

#### Comparison of Pre-surgical Variables

There were 54 patients with Fuwai Cp, with an average age of 49 years of age, of which 29 were male patients (53.7%). There were 832 patients with Fuwai Ct, with an average age of 48 years of age, of which 635 were male patients (76.32%). There were 177 patients with type Cd, with an average age of 49 years of age, of which 154 (87.01%) were male patients. There was no significant difference in the mean age between the three groups, however there were statistically significant differences in the proportion of males in the three groups (Ct vs. Cd = 76.32 vs. 87.01%, *P* < 0.01; Ct vs. Cp = 76.32 vs. 53.07%, *P* < 0.01; Cd vs. Cp = 87.01 vs. 53.07%, *P* < 0.01). The proportion of aortic root aneurysms in patients with type Cd was the lowest (9.6%), showing a statistically significant difference between type Ct and type Cp (*P* < 0.01). The proportion of renal insufficiency and pericardial effusion of type Ct was the highest. Statistical comparisons of the variables before surgery for the various types of dissections were shown in Table 1.

**Table 1:** Comparison of pre-operative variables for the three aortic arch dissections.

**Variable**	**Fuwai Cp** **(*n* = 54)**	**Fuwai Ct** **(*n* = 832)**	**Fuwai Cd** **(*n* = 177)**	***P*** **-value**		
				**Ct vs. Cd**	**Ct vs. Cp**	**Cd vs. Cp**
Age, M (IQR)	49 (19)	48 (16)	49 (17)	>0.03	>0.03	>0.03
Male (%)	29 (53.7)	635 (76.32)	154 (87.01)	<0.01^**^	<0.01^**^	<0.01^**^
Marfan (%)	3 (5.56)	88 (10.59)	11 (6.21)	0.07	0.23	0.85
Diabetes (%)	2 (3.7)	20 (2.4)	3 (1.69)	0.56	0.55	0.37
CAD (%)	5 (9.26)	40 (4.81)	2 (1.13)	0.02	0.14	<0.01^**^
Hypertension (%)	39 (72.22)	610 (73.32)	138 (77.97)	0.20	0.86	0.38
COPD (%)	0 (0)	4 (0.48)	1 (0.56)	0.88	1.00	1.00
Acute phrase(%)	31 (57.41)	591 (71.03)	85 (48.02)	<0.01^**^	0.03	0.22
**Symptom (%)**						
Chest pain	45 (83.33)	731 (87.97)	142 (80.23)	<0.01^**^	0.31	0.61
Back pain	15 (27.78)	264 (31.73)	70 (39.55)	0.04	0.54	0.11
Abdominal pain	12 (22.22)	315 (37.86)	66 (37.29)	0.88	0.02^*^	0.04
Moderate AI or above (%)	19 (35.19)	215 (25.84)	18 (10.17)	<0.01^**^	0.13	<0.01^**^
Aortic root aneurysm (%)	20 (37.04)	233 (28)	17 (9.6)	<0.01^**^	0.15	<0.01^**^
WBC, M (IQR)	9.18 (4.13)	10.68 (5.45)	9.45 (5.87)	<0.01^**^	<0.01^**^	0.34
HB, M (IQR)	129 (31)	132 (26)	139 (26)	<0.01^**^	0.25	<0.01^**^
^**#**^ **PennClassification (%)**						
Pa	48 (88.89)	631 (75.96)	143 (80.79)	<0.01^**^	<0.01^**^	0.04^*^
Pb	3 (5.56)	176 (21.15)	31 (17.52)	<0.01^**^	<0.01^**^	<0.01^**^
Pc	2 (3.7)	17 (2.04)	2 (1.12)	0.03^*^	0.37	0.15
Pbc	1 (1.8)	8 (0.96)	1 (0.56)	<0.01^**^	0.39	0.07
Renal insufficiency (%)	6 (11.11)	137 (16.47)	16 (9.04)	0.01^*^	0.30	0.65
Liver dysfunction (%)	3 (5.56)	40 (4.81)	0 (0)	<0.01^**^	0.80	0.01^*^
Pericardial effusion (%)	5 (9.26)	106 (12.74)	5 (2.82)	<0.01^**^	0.45	0.04

# *Penn Classification: Penn Class a (No ischemia), Penn Class b (Localized ischemia), Penn Class c (Generalized ischemia/circulatory collapse), Penn Class b&c (Combined ischemia). COPD, chronic obstructive pulmonary disease; CAD, coronary artery disease; M, median; IQR, interquartile range*.

* *P < 0.05*,

** *P < 0.01*.

#### Comparison of the Operative Variables

The cardiopulmonary bypass time, clamp time, and hypothermic circulatory arrest time for the type Ct patients were longer compared to type Cp and type Cd (*P* < 0.03). The ratio of partial arch replacement for type Cp was higher compared to type Ct and type Cd (Cp vs. Ct = 62.96 vs. 1.44%, *P* < 0.01; Cp vs. Cd = 62.96 vs. 0, *P* < 0.01). The proportion of total aortic arch replacement performed on type Ct was higher compared to Cp type and Cd type (Ct vs. Cp = 98.56 vs. 37.04%, *P* < 0.01; Ct vs. Cd = 98.56 vs. 19.77%, *P* < 0.01). The highest proportion (70.62%) of thoracic endovascular aortic repair (TEVAR) was performed on type Cd and was statistically different from type Cp and type Ct (*P* < 0.01). The comparisons for operative variables for the various types of dissection are shown in Table 2.

**Table 2:** Comparison of operative variables for the three aortic arch dissections.

**Variable**	**Fuwai Cp** **(*n* = 54)**	**Fuwai Ct** **(*n* = 832)**	**Fuwai Cd** **(*n* = 177)**	***P*** **-value**		
				**Ct vs. Cd**	**Ct vs. Cp**	**Cd vs. Cp**
Blood loss_ml, M (IQR)	600 (315)	700 (600)	100 (550)	<0.01^**^	0.10	<0.01^**^
CPB time_min, M (IQR)	138 (71)	183 (66)	0 (90)	<0.01^**^	<0.01^**^	<0.01^**^
Clamp time_min, M (IQR)	73.5 (49)	94 (38)	0 (28)	<0.01^**^	<0.01^**^	<0.01^**^
HCA time_min, M (IQR)	0 (17)	21 (8)	0 (0)	<0.01^**^	<0.01^**^	0.22
Partial aortic arch replacement (%)	34 (62.96)	12 (1.44)	0 (0)	0.14	<0.01^**^	<0.01^**^
Total arch replacement (%)	20 (37.04)	820 (98.56)	37 (20.91)	<0.01^**^	<0.01^**^	0.01^*^
TEVAR (%)	0 (0)	0 (0)	125 (70.62)	<0.01^**^	1.00	<0.01^**^
Aortic root replacement (%)	20 (37.04)	233 (28)	17 (9.6)	<0.01^**^	0.15	<0.01^**^
CABG (%)	8 (14.81)	82 (9.86)	3 (1.69)	<0.01^**^	0.24	<0.01^**^
CPB (%)	53 (98.15)	828 (99.52)	61 (34.46)	<0.01^**^	0.19	<0.01^**^
HCA (%)	18 (33.33)	724 (87.02)	31 (17.51)	<0.01^**^	<0.01^**^	0.01^*^

*CPB, cardiopulmonary bypass; HCA, hypothermic circulatory arrest; TEVAR, thoracic endovascular aortic repair; CABG, coronary artery bypass grafting; M, median; IQR, interquartile range*.

* *P < 0.05*,

** *P < 0.01*.

#### Comparison of the Postoperative Variables

There was 1 patient (1.85%) who died during type Cp surgery, 78 patients (9.38%) who died during type Ct surgery, and 3 patients (1.69%) who died during type Cd surgery. The mortality rate of type Ct was higher compared to type Cd (*P* < 0.01). The mortality rate of the type Ct was higher compared to the type Cp (*P* = 0.06). There were no significant differences in the mortality rate between type Cd and type Cp (*P* = 0.93). The comparisons for postoperative variables for the various types of dissection are shown in Table 3.

**Table 3:** Comparison of post-operative variables for the three aortic arch dissections.

**Variable**	**Fuwai Cp** **(*n* = 54)**	**Fuwai Ct** **(*n* = 832)**	**Fuwai Cd** **(*n* = 177)**	***P*** **-value**		
				**Ct vs. Cd**	**Ct vs. Cp**	**Cd vs. Cp**
Hospital stay_day, M (IQR)	8 (6)	12 (6)	7 (5)	<0.01^**^	0.10	<0.01^**^
Mechanical ventilation time, M (IQR)	15 (12)	16.5 (16)	13 (12)	<0.01^**^	<0.01^**^	<0.01^**^
Surgical mortality (%)	1 (1.85)	78 (9.38)	3 (1.69)	<0.01^**^	0.06	0.93
Acute kidney injury (%)	12 (22.22)	264 (31.73)	23 (12.99)	<0.01^**^	0.14	0.09
Liver dysfunction (%)	11 (20.37)	265 (31.85)	20 (11.3)	<0.01^**^	0.07	0.08
CRRT (%)	4 (7.41)	86 (10.34)	9 (5.08)	0.03^*^	0.49	0.51
Stroke (%)	2 (3.7)	19 (2.28)	3 (1.69)	0.62	0.51	0.37
Paraplegia (%)	1 (1.85)	36 (4.33)	3 (1.69)	0.10	0.37	0.93
Gastrointestinal bleeding (%)	2 (3.7)	21 (2.52)	2 (1.13)	0.25	0.59	0.20

*CRRT, continuous renal replacement therapy; M, median; IQR, interquartile range*.

* *P < 0.05*,

** *P < 0.01*.

#### Long-Term Follow-Up Comparison

**Figure 3:**
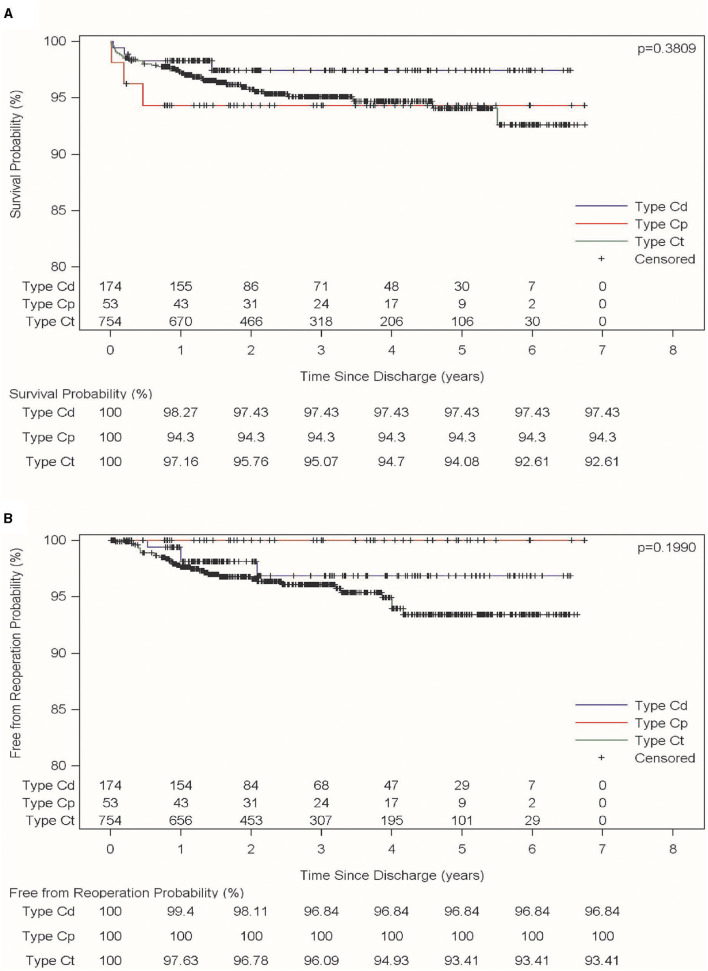
Long-term follow-up for the three different types of aortic arch dissection. **(A)** Referred to long-term survival for the three different types of aortic dissection; **(B)** referred to the long-term absence of aortic-related re-surgeries for the three different types of aortic dissection.

The 5-year survival rates of types Cp, Ct, and Cd were 94.3, 94.08, and 97.43% respectively. There were no significant differences in the long-term survival rates among the three types (*P* = 0.3809). The long-term survival curves for the three types were shown in Figure 3A. During the follow-up period, there were no aorta-related reoperations for patients with Cp type. The 5-year rates free of aorta-related reoperation of type Ct and type Cd were 93.41 and 96.84%, respectively. There were no statistically significant differences in aorta-related reoperations for the three types (*P* = 0.1990; Figure 3B).

### The Distribution of Surgery in Different Classification

**Figure 4:**
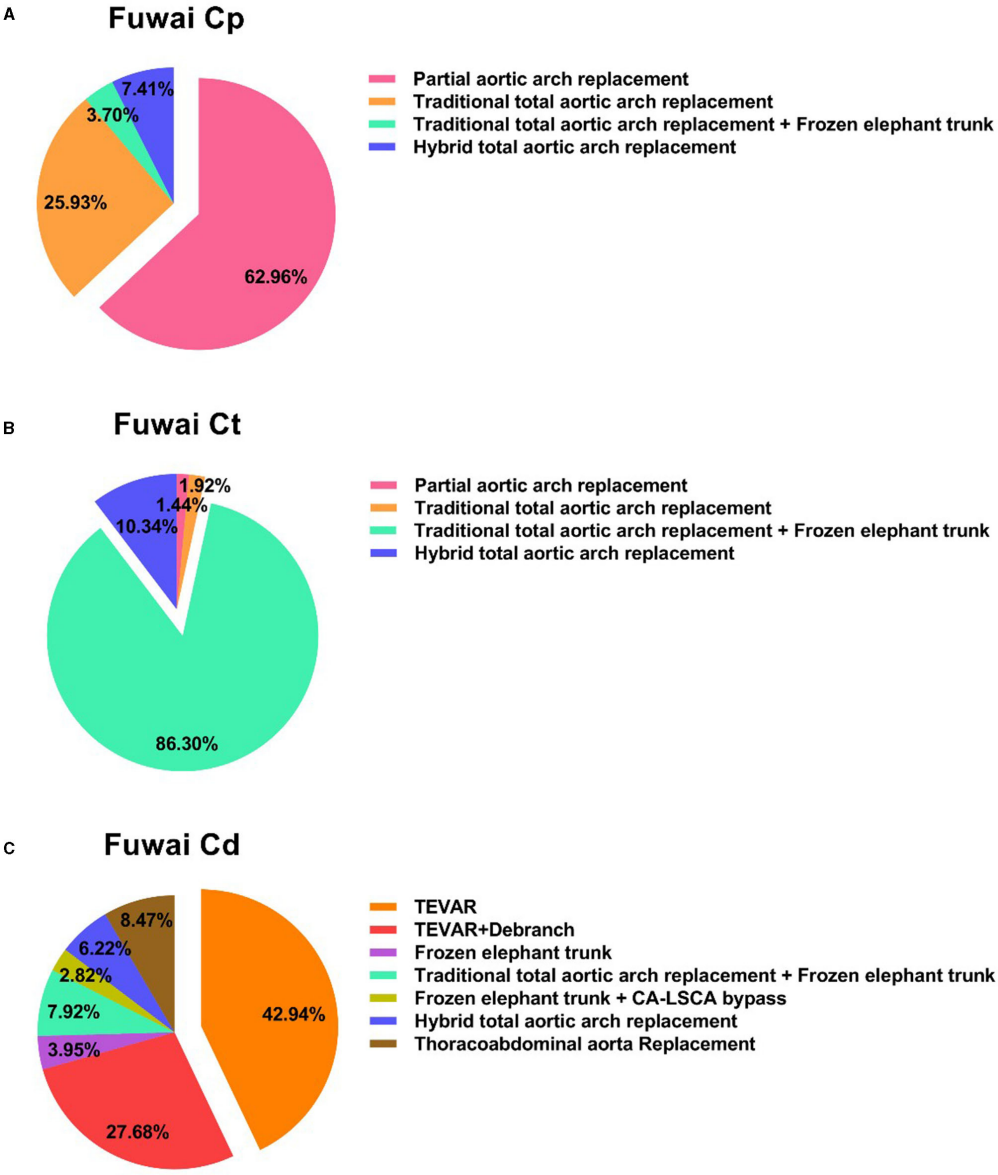
Distribution of surgical procedures for the three different types of dissection. **(A)** Referred to the surgical procedures of Fuwai Cp; **(B)** referred to the surgical procedures of Fuwai Ct; **(C)** referred to the surgical procedures of Fuwai Cd. TEVAR, thoracic endovascular aortic repair; CA-LSCA, carotid artery- left subclavian artery.

For Fuwai type Cp, 34 patients (62.96%) were treated with partial aortic arch replacement, and 20 patients (37.04%) were treated with total arch replacement. There were 13 patients with an aortic ulcer or intramural hematoma in the distal arch (the distal end of the left common carotid artery): these patients received traditional total aortic arch replacement. There were three patients with Marfan syndrome, two patients who received a total arch replacement and frozen elephant trunk (FET), and one patient who received traditional total aortic arch replacement due to a previous thoracoabdominal aortic replacement before admission. There were four patients above 50 years old and who had thoracic aorta lesions, and they eventually received a hybrid total aortic arch replacement. For Fuwai type Ct patients, there were 12 patients with partial aortic arch replacement (1.44%) and 820 patients with total aortic arch replacement (98.56%). There were 12 patients above 70 years of age, for whom partial aortic arch replacement was performed to reduce operative risk. Among 820 total aortic arch replacement patients, including 718 patients who received a traditional total aortic arch replacement and FET for the dissection extended to thoracic aorta, 16 patients received a total aortic arch replacement, and 86 patients received hybrid total aortic arch replacement due to higher operative risk. Of the 177 patients with Fuwai type Cd, 125 (70.62%) patients were treated with TEVAR, including 76 pure TEVAR cases and 49 TEVAR+ debranch cases. There were 15 patients who received thoracoabdominal aortic replacement due to chronic aortic dissection, which meant that the true lumen couldn't be opened by the stent. There were 12 patients who received FET to treated distal arch dissection because they were <40 years old, including 7 pure FET cases and 5 FET plus left common carotid artery-left subclavian artery bypass. There were 25 patients who received a total aortic arch replacement, including 14 patients who received a total arch replacement and frozen elephant trunk because they had proximal aortic arch lesion or connective tissue diseases, and 11 of these hybrid total aortic arch replacements for these patients had higher operative risk. The 11 patients with Marfan syndrome received total arch replacement and FET. The distributions of surgical methods for the various types are shown in Figure 4.

## Discussion

The Fuwai classification is a supplement to the traditional classification system. Debakey summarized the treatment efficacy of aortic dissection and proposed the Debakey classification (8). With the development of surgical techniques, Tsagakis extended the distal involvement of DeBakey type II dissection to the end of the left subclavian artery (9). This classification method highlights the difference of prognosis between proximal aortic dissection and Debakey type I dissection and resolves the naming of the proximal AcD. However, there is still no clear classification method for distal AcD. Though some researchers have classified the distal arch dissection as Stanford B aortic dissection, this may lead to confusion (10). The Fuwai classification is centered on AcD, which would help to resolve the ambiguity of the AcD that is present in the traditional classification methods. The unified classification method could facilitate academic communication and comparisons from different centers. From the study, we found different types of AcD had different clinical characteristics and required different surgical strategies.

This study summarized 1,063 cases of AcD and found that the average age was lower than previously reported in the International Registry of Acute Aortic Dissection (IRAD) (11). The proportion of patients with Marfan syndrome is higher than previous western studies (12), which is consistent with the research results of domestic scholars (13). In this study, it was found that type Cp patients often also had aortic root aneurysms: we speculated these patients had progressed AcD because of their aortic root aneurysm. Type Cd had the lowest proportion of acute stage patients, which may be due to our therapy strategy in which patients received surgery only if conservative treatments were not working. Fuwai Type Ct had the highest proportion of visceral vascular ischemia, indicating that this type of AcD had more severe preoperative situations.

Different types of AcD have different rates of surgical mortality. While the mortality rate of type Ct dissection was higher than that of type Cp, there was no significant statistical difference. This could have been due to the small sample size, and if the sample size was increased, a statistical difference may emerge. Type Cp was primarily treated with partial aortic arch replacement and did not require deep hypothermia arrest during surgery. Hence, the intraoperative extracorporeal circulation time, blocking time, and operation times were significantly shorter compared to type Ct. This may partly explain the reason for the lower mortality rate of type Cp, which is consistent with previous studies (14). The mortality rate of type Cd was the lowest and was significantly different compared to type Ct. This may be due to the different surgical methods or to the difference in the degree of preoperative organ ischemia (12). The IRAD found that there was no statistically significant difference in 30-day mortality and long-term survival between Stanford type B dissection patients with aortic arch involvement and Stanford type B dissection patients without aortic arch involvement (15). Hence the prognosis of type Cd and Stanford type B dissection may be similar, which requires further study. There was no significant difference in surgical mortality between type Cp and type Cd, indicating that the risk of dissection involving the arch was similar between the two types.

Long-term follow-up showed that the 5-year overall survival rate of AcD was 94.7%, which was different from the previous report of aortic dissection of Stanford type A or type B. The previous literature reported that the 5-year survival rates of Stanford type A and B were 85.7 and 83.3%, respectively (16). There was no significant difference in the long-term survival rate of the three types of AcD. Therefore, the current treatment strategies of our center can achieve satisfactory long-term results. As can be seen from the survival curves, the survival curve of type Ct showed a slow decline, while type Cp and Cd type had a significant plateau. Therefore, further follow-up study is needed to determine whether there is any difference in the long-term survival rate. Type Cp was usually cured completely, as we have had no reports of reoperation. There were two patients with Marfan syndrome who received total arch replacement: their descending aorta diameter enlarge slowly and closely monitoring was needed. Type Ct and Cd had some cases of reoperation due to the gradual increase in the diameter of the resident or visceral vascular ischemia, which was consistent with previous studies (17).

Different types of AcD required different operations. The surgical method for aortic dissection primarily depends on the scope of dissection and the pathological features of the aorta (18). For type Cp patients, 37% underwent total aortic arch replacement. This was mainly because the diameter of the distal aorta of the dissection was >50 mm, or present with aortic arch ulcers, aortic intramural hematomas, etc. For type Ct, the total aortic arch replacement was performed in our center, including the traditional and hybrid total arch. Studies have shown that hybrids could be considered for elderly patients to avoid hypothermia arrest and reduce surgical risks (19). For type Ct, there was still controversy regarding treatment strategy (20, 21). In this study, more than 90% of type Ct patients underwent total arch replacement. This was mainly because of the younger age of patients in this group and the higher proportion of patients with connective tissue disease. This was consistent with previously published results (13). For type Cd, clinicians need not only consider conservative treatment but also decide on whether TEVAR or surgery was needed. For type Cd, if the patient has aortic rupture, renal and lower limb ischemia, and persistent pain did not relieve, we performed TEVAR. To increase the anchoring zone, we performed neck incisions or median thoracotomy to reconstruct the left subclavian artery, left common carotid artery, or the anonymous artery (19). 27% of Fuwai Cd patients underwent debranching, which was higher compared to traditional Stanford type B dissection (22). For Marfan patients with type Cd aortic dissection, we performed total arch replacement combined with stent elephant trunk for better long-term intervention.

This study contained some limitations. First of all, for all retrospective observational sources of data, the potential of data collection bias is always an issue. In this study, biases among the enrolled patients who suffered from different underlying medical conditions, and among the different preoperative therapies that may impact the prognosis, were clearly recognized. The enrolment of consecutive patients, objective collection of records, and consistent operative standards minimized the potential bias among patients. Secondly, the Fuwai classification is only based on the involvement range from preoperative CT imaging, as we did not enroll organ ischemic state or hemodynamic, but since the Fuwai classification is mainly to help the selection of surgery, the scope of involvement can determine the operation method. Finally, although this study contained the largest dataset of AcD in China, it requires confirmation by future larger, multi-center prospective studies in the future.

## Conclusions

The Fuwai classification describes the involvement extent of AcD comprehensively and concisely. It addresses the limitations of traditional classifications in the aortic arch. The Fuwai classification helps to distinguish different AcD, which have different surgical mortality rates and operation methods, but they have similar long-term results.

## Data Availability Statement

The original contributions presented in the study are included in the article/Supplementary Material, further inquiries can be directed to the corresponding author/s.

## Ethics Statement

The studies involving human participants were reviewed and approved by the Ethics Committee of Fuwai Hospital (Approval No: 2017-877). Written informed consent was not required due to its observational nature (Reference Number: ChiECRCT-20180041).

## Author Contributions

JQ and CY designed the study. JQ wrote the paper. JQ, XL, JW, WP, QC, XQ, XS, BW, LZ, SL, WJ, and CY organized patient recruitment. JQ and JW were involved in the statistical analyses and diagramming. All author contributed to the article.

## Funding

This study was supported by Beijing Science and Technology Program (Grant No. Z191100007619042), Capital Health Development and Scientific Research Foundation (Grant No. 2018-2-4035), CAMS Initiative for Innovative Medicine (Grant No. 2016-I2M-1-016), and Special Subject Development Foundation of Fuwai Hospital (Grant No. 2015-FWTS01).

## Conflict of Interest

The authors declare that the research was conducted in the absence of any commercial or financial relationships that could be construed as a potential conflict of interest.

## Publisher's Note

All claims expressed in this article are solely those of the authors and do not necessarily represent those of their affiliated organizations, or those of the publisher, the editors and the reviewers. Any product that may be evaluated in this article, or claim that may be made by its manufacturer, is not guaranteed or endorsed by the publisher.

## References

[1] Vilacosta I San Román JA. Acute aortic syndrome. Heart. (2001) 85:365–8. 10.1136/heart.85.4.365 11250953 PMC1729697

[2] Vilacosta I Aragoncillo P Cañadas V San Román JA Ferreirós J Rodríguez E. Acute aortic syndrome: a new look at an old conundrum. Heart. (2009) 95:1130–9. 10.1136/hrt.2008.153650 19131440

[3] Golledge J Eagle KA. Acute aortic dissection. Lancet. (2008) 372:55–66. 10.1016/S0140-6736(08)60994-0 18603160

[4] von Segesser LK Killer I Ziswiler M Linka A Ritter M Jenni R. Dissection of the descending thoracic aorta extending into the ascending aorta. A therapeutic challenge. J Thorac Cardiovasc Surg. (1994) 108:755–61. 10.1016/S0022-5223(94)70304-3 7934113

[5] Poon SS Theologou T Harrington D Kuduvalli M Oo A Field M. Hemiarch versus total aortic arch replacement in acute type A dissection: a systematic review and meta-analysis. Ann Cardiothorac Surg. (2016) 5:156–73. 10.21037/acs.2016.05.06 27386403 PMC4893527

[6] Rudarakanchana N Jenkins MP. Hybrid and total endovascular repair of the aortic arch. Br J Surg. (2018) 105:315–27. 10.1002/bjs.10713 29488648

[7] Yu CT. The considerations of surgical treatment strategies of acute type A aortic dissection. J Thorac Cardiovasc Surg. (2016) 152:935–7. 10.1016/j.jtcvs.2016.04.027 27530644

[8] DeBakey ME Henly WS Cooley DA Morris GC Jr Crawford ES Beall AC Jr. Surgical management of dissecting aneurysms of the aorta. J Thorac Cardiovasc Surg. (1965) 49:130–49. 10.1016/S0022-5223(19)33323-9 14261867

[9] Tsagakis K Tossios P Kamler M Benedik J Natour D Eggebrecht H. The DeBakey classification exactly reflects late outcome and re-intervention probability in acute aortic dissection with a slightly modified type II definition. Eur J Cardiothorac Surg. (2011) 40:1078–84. 10.1016/j.ejcts.2011.03.037 21570858

[10] Lempel JK Frazier AA Jeudy J Kligerman SJ Schultz R Ninalowo HA. Aortic arch dissection: a controversy of classification. Radiology. (2014) 271:848–55. 10.1148/radiol.14131457 24617732

[11] Tsai TT Trimarchi S Nienaber CA. Acute aortic dissection: perspectives from the international registry of acute aortic dissection (IRAD). Eur J Vasc Endovasc Surg. (2009) 37:149–59. 10.1016/j.ejvs.2008.11.032 19097813

[12] Di Eusanio M Trimarchi S Patel HJ Hutchison S Suzuki T Peterson MD. Clinical presentation, management, and short-term outcome of patients with type A acute dissection complicated by mesenteric malperfusion: observations from the international registry of acute aortic dissection. J Thorac Cardiovasc Sur. (2013) 145:385–90. 10.1016/j.jtcvs.2012.01.042 22341418

[13] Wang W Duan W Xue Y Wang L Liu J Yu S. Clinical features of acute aortic dissection from the registry of aortic dissection in China. J Thorac Cardiovasc Surg. (2014) 148:2995–3000. 10.1016/j.jtcvs.2014.07.068 25433882

[14] Leontyev S Borger MA Etz CD Moz M Seeburger J Bakhtiary F. Experience with the conventional and frozen elephant trunk techniques: a single-centre study. Eur J Cardiothorac Surg. (2013) 44:1076–83. 10.1093/ejcts/ezt252 23677901

[15] Tsai TT Isselbacher EM Trimarchi S Bossone E Pape L Januzzi JL. Acute type B aortic dissection: does aortic arch involvement affect management and outcomes? Insights from the International Registry of Acute Aortic Dissection (IRAD). Circulation. (2007) 116:I150–6. 10.1161/CIRCULATIONAHA.106.681510 17846296

[16] Howard DP Banerjee A Fairhead JF Perkins J Silver LE Rothwell PM. Population-based study of incidence and outcome of acute aortic dissection and premorbid risk factor control: 10-year results from the oxford vascular study. Circulation. (2013) 127:2031–7. 10.1161/CIRCULATIONAHA.112.000483 23599348 PMC6016737

[17] Isselbacher EM. Epidemiology of Thoracic Aortic Aneurysms, Aortic Dissection, Intramular Hematoma, and Penetrating Atherosclerotic Ulcers. New York, NY: Springer Science (2007). 10.1007/978-0-387-36001-0_1

[18] Thrumurthy SG Karthikesalingam A Patterson BO Holt PJ Thompson MM. The diagnosis and management of aortic dissection. BMJ. (2011) 344:d8290. 10.1136/bmj.d8290 22236596

[19] Chang Q Tian C Wei Y Qian X Sun X Yu C. Hybrid total arch repair without deep hypothermic circulatory arrest for acute type A aortic dissection (R1). J Thorac Cardiovasc Surg. (2013) 146:1393–8. 10.1016/j.jtcvs.2012.09.041 23142116

[20] Shi E Gu T Yu Y Yu L Wang C Fang Q. Early and midterm outcomes of hemiarch replacement combined with stented elephant trunk in the management of acute DeBakey type I aortic dissection: comparison with total arch replacement. J Thorac Cardiovasc Surg. (2014) 148:2125–31. 10.1016/j.jtcvs.2013.10.058 24290707

[21] Easo J Weigang E Hölzl PP Horst M Hoffmann I Blettner M. Influence of operative strategy for the aortic arch in DeBakey type I aortic dissection: analysis of the German registry for acute aortic dissection type A. J Thorac Cardiovasc Surg. (2012) 144:617–23. 10.1016/j.jtcvs.2011.07.066 22099946

[22] Zhu J Xi EP Zhu SB Yin GL Wang RP Zhang Y. Management of the vertebral artery during thoracic endovascular aortic repair with coverage of the left subclavian artery. J Thorac Dis. (2017) 9:1273–80. 10.21037/jtd.2017.04.27 28616278 PMC5465137

