# Supplementary figures and images for: A New Aortic Arch Dissection Classification: The Fuwai Classification
Source: Front Cardiovasc Med. 2021 Sep 14;8:710281. doi: 10.3389/fcvm.2021.710281 (PMC8476803; doi:10.3389/fcvm.2021.710281)

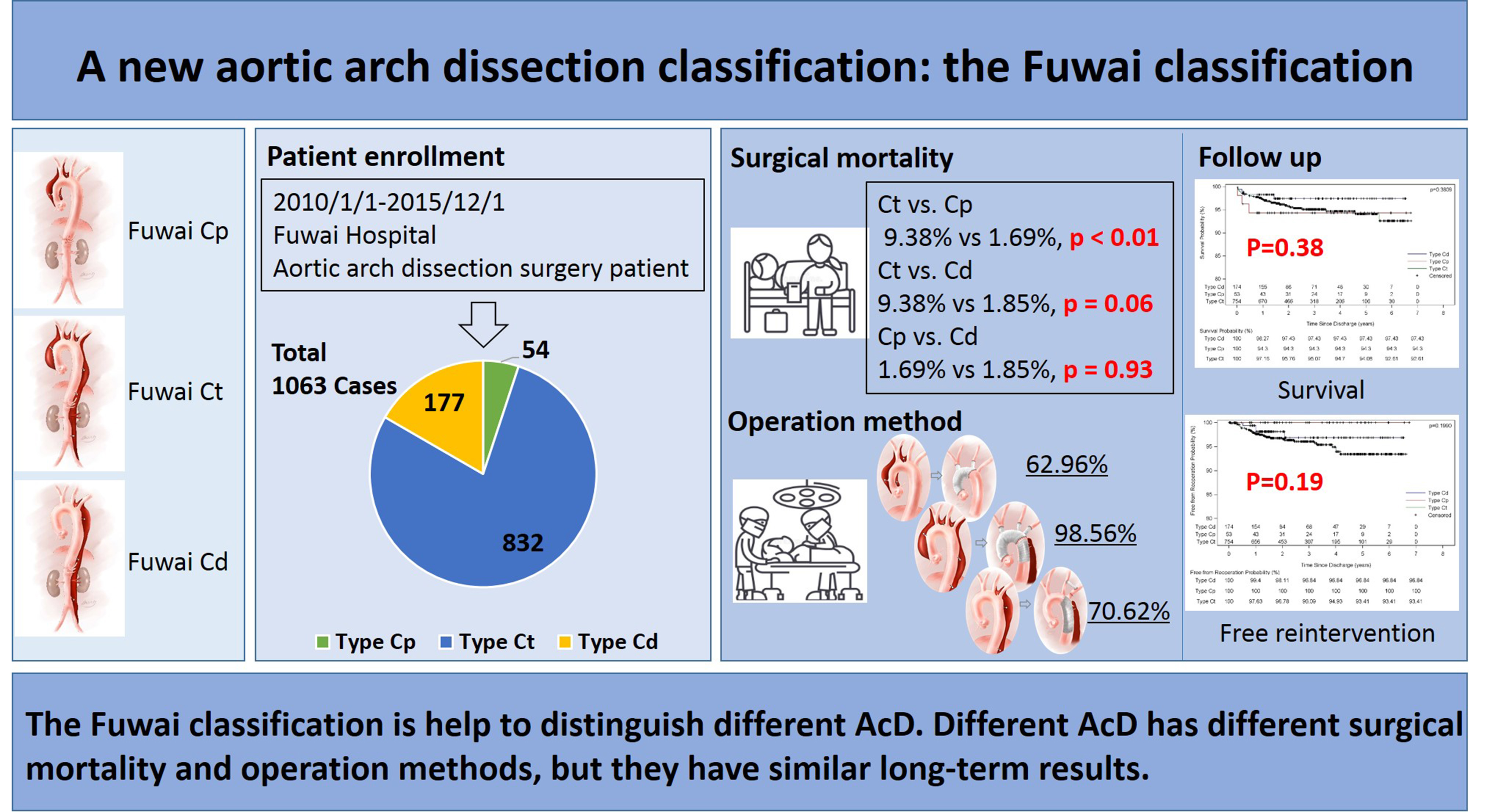

Supplement: Supplementary file 1 [file Image_1.JPEG]

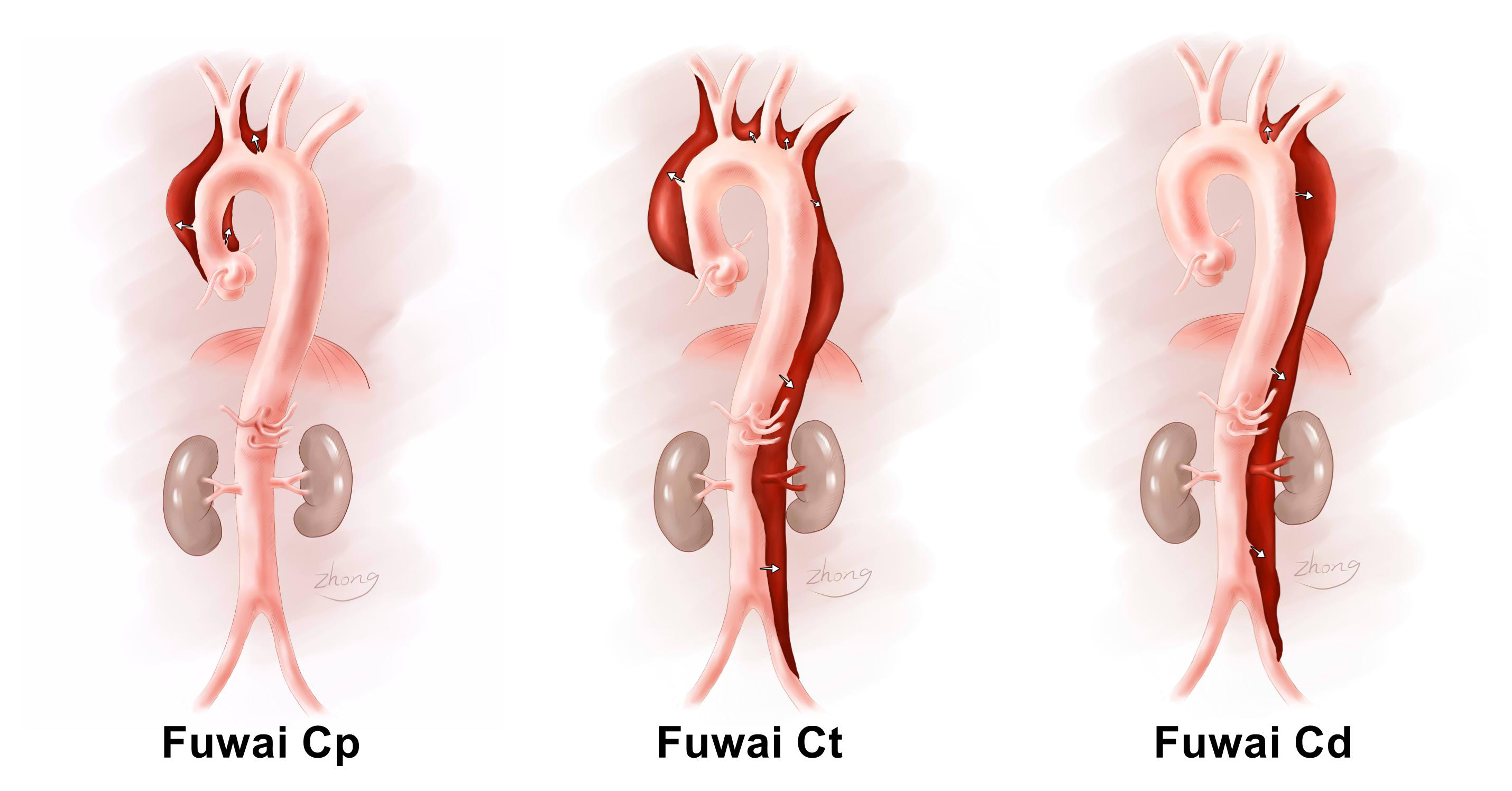

Supplement: Supplementary file 2 [file Image_2.jpg]
